# Clinical study of harvesting lymph nodes with carbon nanoparticles in advanced gastric cancer: a prospective randomized trial

**DOI:** 10.1186/s12957-016-0835-3

**Published:** 2016-03-24

**Authors:** Ziyu Li, Sheng Ao, Zhaode Bu, Aiwen Wu, Xiaojiang Wu, Fei Shan, Xin Ji, Yan Zhang, Zhaodong Xing, Jiafu Ji

**Affiliations:** Key Laboratory of Carcinogenesis and Translational Research Ministry of Education, Department of Gastrointestinal Surgery, Peking University Cancer Hospital and Institute, Fu-Cheng-Lu Street, Beijing, People’s Republic of China; Department of Medical Statistics, Peking University Cancer Hospital and Institute, Fu-Cheng-Lu Street, Beijing, People’s Republic of China; Department of Gastrointestinal Surgery, Peking University Shenzhen Hospital, Lian-Hua-Lu Street, Shenzhen, People’s Republic of China; Department of Gastrointestinal Surgery, Peking University Cancer Hospital and Institute, Beijing, People’s Republic of China

**Keywords:** Carbon nanoparticles, Advanced gastric cancer, D2 gastrectomy, LN harvesting

## Abstract

**Background:**

The objective of this study is to evaluate the efficiency and safety of carbon nanoparticles (CNPs) for harvesting lymph nodes (LNs) in cases of advanced gastric cancer (AGC).

**Methods:**

Patients with previously untreated resectable AGC were eligible for inclusion in this study. All patients were randomly allocated to two subgroups. In the experimental group, 1.0 mL of CNP was injected into the subserosa of the stomach around the tumor before gastrectomy with D2 dissection. The same procedure was performed directly without any coloring material in the control arm. Following surgery, LNs were harvested, colored LNs were counted, and the diameters were measured by the investigator and pathologist.

**Results:**

Thirty patients were enrolled in the study. We observed no serious adverse effects related to CNP injection. The rate of stained LNs was 46.6 %. The mean number of harvested LNs was larger in the experimental than in the control group (38.33 vs 28.27, *p* = 0.041). A smaller diameter of LNs was recorded in the experimental arm (3.32 vs 4.30 mm, *p* = 0.023). In addition, we developed a model for predicting the total number of LNs based on the data from CNP-stained LNs and metastatic LNs (MLNs).

**Conclusions:**

CNP is a safe material. Surgeons could harvest more LNs in patients with AGC. The harvest of an increased number of smaller diameters of LNs may be beneficial. Further study is warranted to demonstrate the model’s practicality.

## Background

Although gastric cancer decreased from being the most common cancer in 1975 to being the fifth most common neoplasm in 2012, it remains the third leading cause of cancer death worldwide, contributing to 723,000 deaths annually [1, 2]. Screening and broad-based awareness of the disease has improved the identification rates of early-stage cancers and superior survival. However, compared with some developed countries, such as Japan, a majority of patients are diagnosed with advanced gastric cancer (AGC) in China, which presents a treatment challenge.

Gastrectomy with D2 lymph node (LN) dissection is the standard treatment for AGC in Asia because of the survival benefit and low complication rate [3, 4]. A similar study result was published recently with data from western countries [5]. There was no controversy on the necessity of dissecting lymph nodes. In the light of guidelines, histopathological examination of at least 15 regional lymph nodes is necessary to accurately assign the N category for gastric carcinoma. Intriguingly, undoubtedly reflecting the contribution of stage migration and dissecting more metastatic LNs, representing the quality of the operation, overall survival (OS) improved incrementally with higher LN counts [6–8]. Based on these results, we attempted to develop a method to obtain more LNs. In other studies, lymphatic tracers, including dye materials, have been used to meet this need [9]. Carbon nanoparticles (CNPs) are a practicable material for harvesting lymph nodes in our department.

CNPs with a mean size of 150 nm can be taken up selectively by the lymphatics after injection into the tissue. The draining regional lymph nodes are thereby colored black, which may provide guidance to the surgeon during lymph node dissection and help harvest lymph nodes after surgery, especially smaller LNs. However, there is insufficient evidence to justify its efficacy for those purposes. Therefore, we carried out a prospective randomized controlled trial on lymph node vital staining for LN dissection and harvesting in AGC.

## Methods

### Patients

**Fig. 1:**
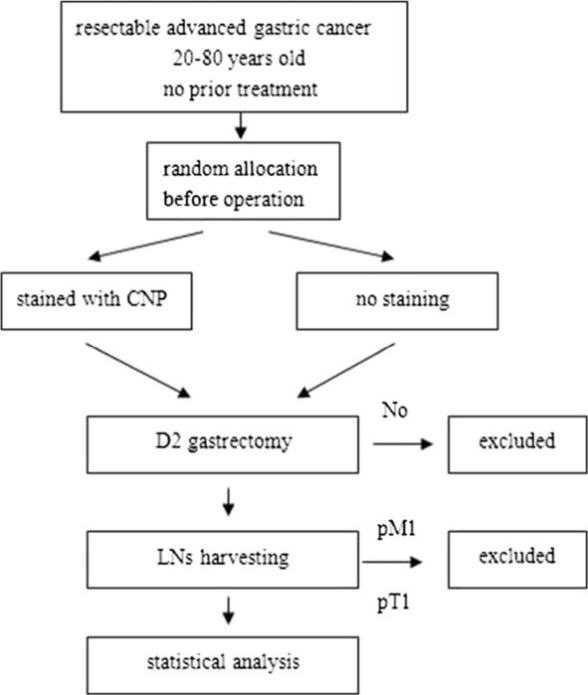
Trial scheme

Thirty-two 20- to 80-year-old resectable AGC patients from December 2013 to June 2014 diagnosed by pathological biopsy, staged by computed tomography (CT) and endoscopic ultrasonography (EUS), who had no prior treatment, were chosen for this trial. All patients were diagnosed by a multi-disciplinary team and provided written informed consent before surgery. The exclusion criteria were as follows: R0 resection was not achieved by gastrectomy with D2 lymphadenectomy according to Japanese gastric cancer treatment guidelines 2010 (ver.3) [10], having an allergic reaction, being pregnant, or being proved to be stage T1 or M1 after surgery. Two patients were excluded; one was pathologically diagnosed as in the T1 stage and the other was cytology positive by a laparoscopic approach. Thus, 30 patients were analyzed. Figure 1 shows the trial scheme.

### CNP staining, open gastrectomy with D2 dissection, and lymph node harvesting

**Fig. 2:**
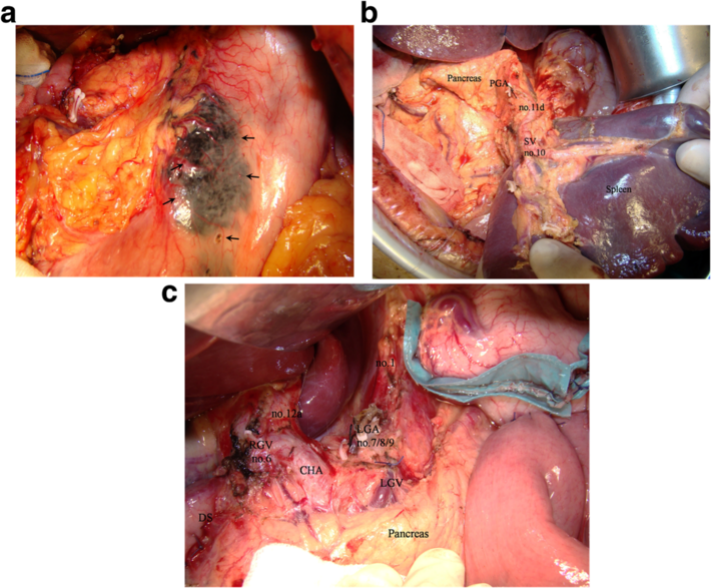
**a** Carbon nanoparticles were injected into the subserosa of the stomach around the tumor; the *arrows* show the injection sites. **b** D2 dissection performed in total gastrectomy; spleen-preserving station no. 10 was resected. *SV* splenic vessels, *PGA* posterior gastric artery. **c** D2 dissection performed in distal gastrectomy; portions of dissected LNs are shown. *CHA* common hepatic artery, *LGA* left gastric artery, *LGV* left gastric vein, *RGV* right gastric vessels, *DS* duodenal stump.

The patients were enrolled before the operation according to a randomized table generated by the statistician (Yan Zhang). All were randomly allocated to two subgroups. In the experimental group, 1.0 mL of CNP (carbon nanoparticles suspension injection, 1 mL/50 mg, Chongqing, China) was injected into the subserosa of the stomach at five points around the tumor on average (0.2 mL in each cardinal point adjacent to the lesion, Fig. 2a) 10 min before open gastrectomy with D2 dissection. In distal gastrectomy (DG, Fig. 2c), a free proximal margin of at least 4 cm was necessary according to the gastric cancer treatment guidelines of the Japanese Gastric Cancer Association (ver. 3). En bloc excision was done in lymph nodes of station nos. 1, 3, 4sb, 4d, 5, 6, 7, 8, 9, 11p, 12a, and 14v if metastasis was highly suspected in no. 6. In addition, nos. 2, 11d, and 10 were resected in total gastrectomy (TG, Fig. 2b). The same gastrectomy was performed directly without any coloring material in the control arm. Pictures or videos of the surgery were evaluated by the entire team in our department after the operation to ensure that a standard D2 gastrectomy was performed.

After surgery, the investigator (Sheng Ao) harvested the lymph nodes with the pathologists (10 min for each patient’s specimens) and simultaneously counted the colored LNs. The tissues were fixed in formalin solution and embedded in paraffin for histological examination with H&E staining. Then, the diameters of each LN were measured.

This study was designed as a single-center prospective clinical trial. The procedure was approved by the Ethics Committee of Beijing Cancer Hospital. The study protocol was released on ClinicalTrials.gov (ID: NCT02123407).

### Study design

The primary outcome measure was to calculate the number of harvested LNs. The second outcome measure was the diameter of the harvested LNs, which was aimed to obtain the maximum dimension to reflect the degree of difficulty of picking up LNs. In addition, operation time, bleeding, and complications were compared between the two groups to confirm the safety of CNP.

Respectively, the average number of lymph nodes we harvested in advanced gastric cancer cases without CNP was 28.76 ± 1.14. The sample size was set at 30 (15 each group) based on the assumption that the expected number of LNs should increase by at least one, with a two-sided alpha of 5 % and at least 90 % power. The planned duration of accrual was 7 months. All statistical analyses were conducted with SPSS 17.0 software.

## Results

The background factors, perioperative outcomes, pathological findings, and operation-related outcomes were shown in Table 1. Age, BMI, location, surgery, and pathological stage of tumor were not significantly different in the two groups, nor were the operation time, bleeding, and complications. No allergies and no toxic reactions or side effects from the injection of CNP were recorded in any case.

**Table 1:** Clinical characteristics of patients

Variable	Pattern group	Experimental group	Control	*p* value*
Sex	Male	10 (9.5)	9 (9.5)	0.705
	Female	5 (5.5)	6 (5.5)	
Age (years)	>70	2 (1.5)	1 (1.5)	
	50–70	11 (9.5)	8 (9.5)	0.380
	<50	2 (4.0)	6 (4.0)	
BMI		25.45 ± 3.60	23.80 ± 2.44	0.155
Lauren type	Intestinal	3 (3.0)	3 (3.0)	
	Nonintestinal	12 (12.0)	12 (12.0)	1.000
Location	Upper	5 (4.5)	4 (4.5)	
	Middle	2 (3.0)	4 (3.0)	0.788
	Lower	8 (7.5)	7 (7.5)	
Surgery	DSG	7 (7.0)	7 (7.0)	
	TG	8 (8.0)	8 (8.0)	1.000
Stage	IB	0 (1.0)	2 (1.0)	
	II	5 (6.0)	7 (6.0)	0.242
	III	10 (8.0)	6 (8.0)	
Operation time (min)		214.7 ± 42.9	212.9 ± 55.7	0.922
Bleeding (mL)		110.0 ± 63.2	98.7 ± 66.2	0.635
Complication	Yes	5 (4.0)	3 (4.0)	
	No	10 (11.0)	12 (11.0)	0.682

*Analyzed by Student’s *t* test or the *χ* ^2^ test

**Fig. 3:**
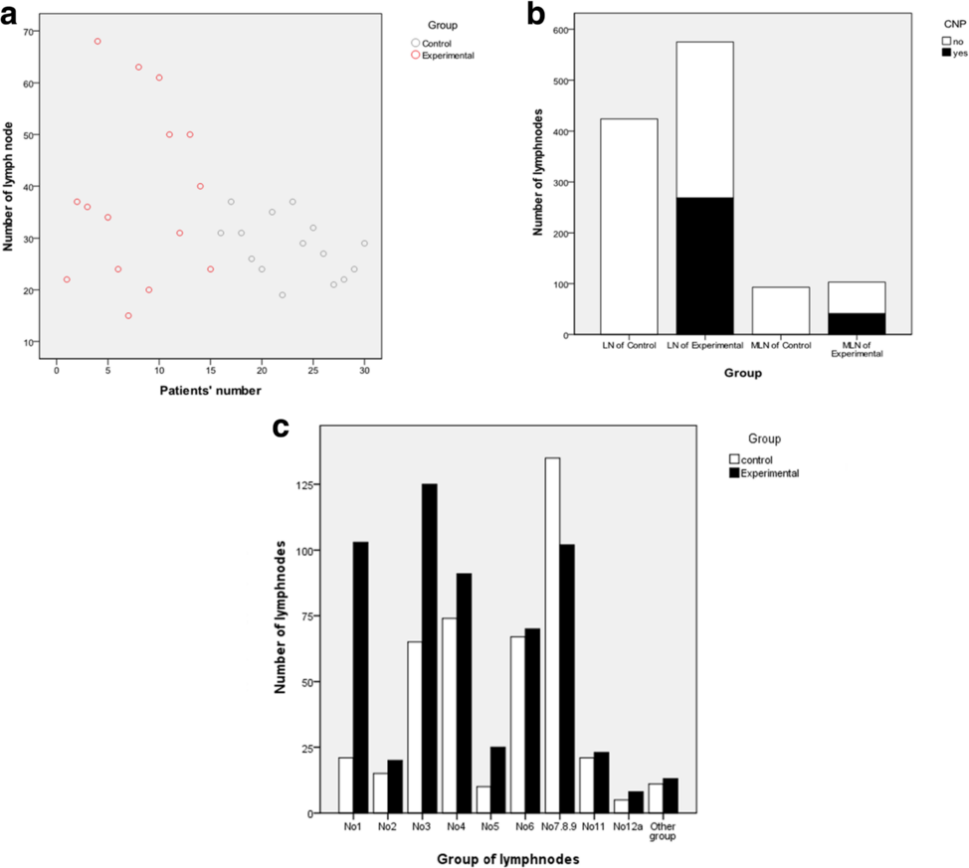
**a** Harvested LNs from every patient. **b** Total number of harvested LNs and metastatic LNs in the experimental and control groups. **c** Harvested LNs from every station

**Fig. 4:**
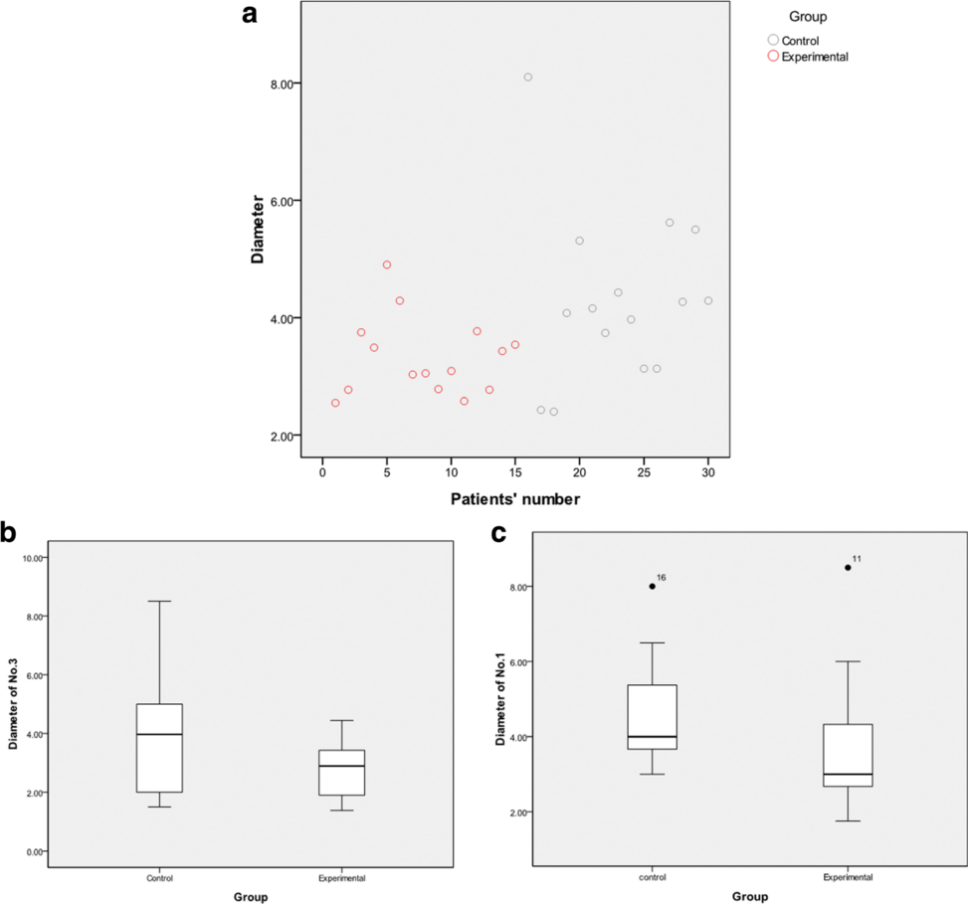
**a** Mean diameters of LNs in each patient. **b** Mean diameter of LNs in station no. 3. **c** Mean diameter of LNs in station no. 1

The harvested LNs of every patient and every station were described in Fig. 3a, c, where significant differences could be found among station nos. 1 and 3 in the two groups. The rate of stained LNs was 46.6 % in the experimental arm (Fig. 3b). The mean number of harvested LNs was larger in the experimental than in the control group (38.33 vs 28.27, *p* = 0.041, Table 2). No significant differences were found on the presence of skip metastases among two groups (0/15 vs 0/15). The ratio of the LN metastasis-positive patients was higher than that of the control group (14/15 vs 7/15, *p* = 0.014), while the number of metastatic LNs (MLNs) was not different (*p* = 0.126, Fig. 3b). To determine why the number of LNs was larger with CNP treatment, additional exploratory analyses of LN diameters were performed (Fig. 4a). In the experimental group, pathological LN diameters ranged from 0.5 to 15.0 mm, with a mean of 3.32 mm. Not unexpectedly, a mean diameter of 4.30 mm with a range from 0.5 to 20.0 mm was recorded in the control group (Table 3). A smaller diameter in station nos. 1 and 3 could be observed in the experimental arm, although this was not statistically significant (Fig. 4b, c).

**Table 2:** Mean number of harvested LNs

	Number	Means of lymph nodes	*p* value
Experimental group	15	38.33	
Control group	15	28.27	0.041

**Table 3:** Mean diameter of harvested LNs

	Number	Means of diameter (mm)	*p* value
Experimental group	15	3.32	
Control group	15	4.30	0.023

We attempted to design a model for predicting the total number of LNs based on the data of CNP-staining LNs and MLNs by utilizing statistical linear regression model method (Table 4). This model proved to be the following linear equation:

$$ Y=11.628+1.062X1+1.126X2 $$

**Table 4:** Model for predicting total number of LNs

Model	*β*	SE	*t*	*p*
Constant^a^	11.628	7.839	1.483	0.164
CNP-staining LNs	1.062	0.329	3.223	0.007
Metastatic LNs	2.373	1.126	0.475	0.035
*R* square		0.537		
Sample size		15		

Dependent variable: number of lymph nodes (*Y*); predictors: constant, CNP-staining LNs (*X*1), metastatic LNs (*X*2)

^a^Gender, age, BMI, location, surgery, operation time, bleeding, diameter, and pathological stage were included

## Discussion

Lymphatic tracers, such as methylene blue, indocyanine green, and an intraoperative radiation technique with a gamma probe, had been widely used as guidance for lymph node searching and dissection for some years [11, 12]. It was initially confirmed that lymphatic tracers were useful to improve OS by harvesting more LNs. In fact, both surgeons and pathologists have trouble harvesting LN because of their busy workload, especially in China, which has the highest number of people in the world. Furthermore, no ideal materials were found due to the limitation of their staining efficiency, the relatively complicated lymphatic flow of the gastric system, radiation injury, and expense. Carbon particles, which are convenient, inexpensive, and widely available in general hospitals, have been used by others for LN staining and were shown to be safe [13, 14]. Similarly, in our study, no CNP-related side effects occurred, there was no extra bleeding, the length of surgery was not extended, and no extra complications were observed.

This prospective randomized controlled trial was mainly designed to test the efficiency of CNP. In the design research process, injection method was considered. Because lymphatic vessels are connected to each other by a communicating branch in the gastric wall which expands vertically, many researchers held the view that injection methods were equally efficient in lymph node staining in AGC [15, 16]. We obtained similar results in a preliminary experiment; thus, we selected to perform subserosal dye injection, which is an easier and more effective method during open surgery. Our staining rate was 46.6 %, somewhat lower than the rate reported in Japanese studies about early gastric cancer [17]. There might be two reasons for this difference. First, more lymphatic vessels were blocked by the tumor in AGC. On the other hand, more LNs from the N2 station were dissected in AGC for the development of gastric surgery, which were more difficult to stain because of distance and lymphatic vessel complex structure.

Obviously, more LNs were harvested in patients with CNP staining (38.33 vs 28.27). The difference agreed with Catarci’s finding using CH40 (a type of dye material) [18]. Nevertheless, we obtained a larger number of LNs and better standard gastrectomy with D2 dissection. However, subgroup analysis showed no more MLNs were harvested in the experimental group, which might be due to sample size. On the other hand, the higher ratio of the LN metastasis-positive patients than that of the control group indicated that CNP could help improve the discovery rate of metastatic LN, but more study are needed to prove the result.

**Fig. 5:**
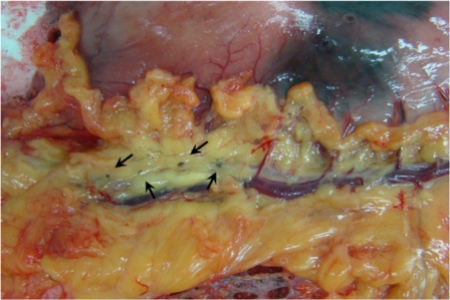
Small LNs were stained by carbon nanoparticles so that they would not be left in case of micrometastases (*arrows* show the small LNs)

To explore why we could harvest more LNs with CNP, we measured the maximum diameter of each LN for a satisfactory outcome. Only we offer the data in detail. From the point of view of the operation, en bloc excision of LNs could be complete because they were distinguished more easily from adipose tissues if colored black. In addition, some small LNs (<2.0 mm) would not be left (Fig. 5) in the case of micrometastases, which could have a very important influence on N staging and treatment strategies. We also realized that the greatest difference happened in station nos. 1 and 3. Although not statistically significant, the clinical value should not be ignored because more small LNs were harvested. Meanwhile, bias in our subgroup analyses was unavoidable so that more studies should be designed to provide such data of every LN station.

Finally, we set up a forecasting model to evaluate the quality of the operation if CNP was used. Since the importance of statistics was noticed, more and more scientists, such as Gretschel [19], have combined it with medicine to solve clinical problems. To some extent, our model may help the surgeons review their work of removing LNs to improve their surgical skills. For instance, surgeons could compare their resected LNs to the predicting number calculated with this model to evaluate the quality of operation. However, this work should be tested in more cases.

## Conclusions

Our results indicated that CNP was a safe material and that surgeons could harvest more LNs with it in cases of AGC, which might benefit from the harvest of an increased number of smaller LNs. The model we built in this study could play a role in evaluating the surgeon’s capacity and their training. However, further study is needed to prove its practicality.
